# Versorgung von rheumatologischen Patienten während des Lockdowns im Frühjahr 2020: Telemedizin, Delegation, Patientenzufriedenheit und Impfverhalten

**DOI:** 10.1007/s00393-021-01005-3

**Published:** 2021-05-11

**Authors:** Thea Thiele, Sonja Beider, Henrik Kühl, Gudrun Mielke, Anna Holz, Stefanie Hirsch, Torsten Witte, Kirsten Hoeper, Anne Cossmann, Christine Happle, Alexandra Jablonka, Diana Ernst

**Affiliations:** 1 Klinik für Rheumatologie und Immunologie, Medizinische Hochschule Hannover, Carl-Neuberg-Str. 1, 30625 Hannover, Deutschland; 2 Rheumatologische Facharztpraxis, Hildesheim, Deutschland; 3 Regionales kooperatives Rheumazentrum Niedersachsen e. V., Hannover, Deutschland; 4 Klinik für Pädiatrische Pneumologie, Allergologie und Neonatologie, Medizinische Hochschule Hannover, Hannover, Deutschland

**Keywords:** Telemedizin, COVID-19, Assistenzberufe, Versorgungsforschung, Pandemie, Telemedicine, COVID-19, Assistant for rheumatology, Healthcare research, Pandemic

## Abstract

**Hintergrund:**

In der ambulanten Versorgung wurde Telemedizin im Lockdown von März bis Mai 2020 eingesetzt. Ziel der Studie war es, Patienten aus einer Praxis und der Hochschulambulanz bezüglich Patientenzufriedenheit mit Telemedizin, COVID-19-Sorgen und Impfverhalten auszuwerten sowie die Gesprächsführung durch eine rheumatologische Fachassistenz (RFA) mit einem Arzt zu vergleichen.

**Methoden:**

Patienten mit rheumatoider Arthritis, Psoriasisarthritis oder Spondyloarthritis ohne Therapieänderung seit der letzten Vorstellung wurde ein telemedizinischer Ersatztermin im Rahmen dieser Studie bei Terminabsage durch die versorgenden Zentren angeboten. Randomisiert wurden sie von einem Arzt oder einer RFA (RFA nur Universität) telemedizinisch versorgt. Die Anamnese erfolgte telefonisch standardisiert mittels Fragebogen. Die Krankheitsaktivität wurde mittels modifizierten Clinical Disease Activity Score (CDAI) und BASDAI festgestellt. Im Anschluss erhielten die Patienten einen pseudonymisierten Evaluationsbogen.

**Ergebnisse:**

Von 112/116 (96 %) eingeschlossenen Patienten schickten 88/112 (79 %) den Fragebogen zurück. RFAs führten 19/112 (17 %) Telefonate. Die Therapie wurde in 19/112 (17 %) geändert. Die meisten Sorgen bezüglich COVID-19 hatten Patienten mit der höchsten Krankheitsaktivität (*p* = 0,031), den meisten schmerzhaften Gelenken (*p* = 0,001) sowie den meisten Schmerzen (VAS Score ≥7) (*p* = 0,009). Diese Patienten hätten auch selbst ihren Termin abgesagt (*p* = 0,015). Die Patientenzufriedenheit mit der Gesprächsführung war gut (Mittelwert 4,3/5,0 modifizierter FAPI), unabhängig von der Institution, der Gesprächsdauer oder dem Gesprächspartner. Patienten mit hoher Schmerzintensität waren am unzufriedensten (*p* = 0,036); 42/100 (38,2 %) Patienten waren gegen Pneumokokken und 59/100 (53,6 %) gegen Influenza geimpft.

**Zusammenfassung:**

Für ausgewählte Patienten ist die telemedizinische Versorgung im Rahmen eines Telefongespräches gut geeignet. Hinsichtlich der Patientenzufriedenheit ist die Delegation einer telefonischen Visite an eine RFA möglich. Bezüglich des Impfverhaltens besteht Verbesserungsbedarf.

**Zusatzmaterial online:**

Die Online-Version dieses Beitrags (10.1007/s00393-021-01005-3) enthält die Studienfragebögen. Beitrag und Zusatzmaterial stehen Ihnen auf www.springermedizin.de zur Verfügung. Bitte geben Sie dort den Beitragstitel in die Suche ein, das Zusatzmaterial finden Sie beim Beitrag unter „Ergänzende Inhalte“.
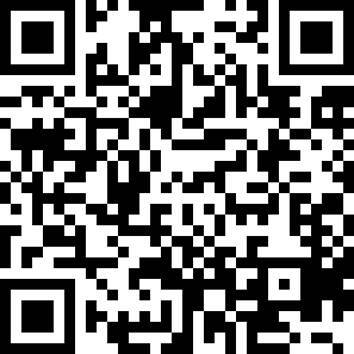

## Abstract (en)

**Background:**

Telemedicine was implemented in outpatient care during the lockdown between March and May 2020. The aim of the study was to assess patients from a private practice and the university outpatient department with respect to patient satisfaction with telemedicine, COVID-19 worries and vaccination behavior and to compare the teleconsultation by a medical assistant for rheumatology (RFA) and a physician.

**Methods:**

Patients with rheumatoid arthritis, psoriatric arthropathy or spondylarthritis without treatment modifications since the previous presentation were offered a telemedical replacement appointment within the framework of this study in the case of appointment cancellation by the treating center. Participants were randomized to a telemedicine appointment by a physician or an RFA (RFA university only). The patient history was carried out by telephone and standardized using a questionnaire. The disease activity was determined using the modified clinical disease activity score (CDAI) and the BASDAI. Subsequently, all patients received a pseudonymized evaluation questionnaire.

**Results:**

In total 112/116 (96%) patients participated. Of these 88/112 (79%) returned the questionnaire. The RFAs conducted 19/112 (17%) of the telephone calls. The treatment was modified in 19/112 (17%) patients. Concerns about contracting COVID-19 correlated with high disease activity (*p* = 0.031) including the presence of painful joints (*p* = 0.001) and high pain levels (VAS ≥7, *p* = 0.009). These patients would have also cancelled their appointment themselves (*p* = 0.015). Patient satisfaction with the consultation was good (mean 4.3/5.0 modified FAPI) independent of the institution, the duration of the consultation and the consultation partner. Patients with a high pain intensity were the least satisfied (*p* = 0.036). Only 42/100 (38.2%) of the patients had been vaccinated against pneumococci and 59/100 (53.6%) against influenza.

**Conclusion:**

Telemedical care within the framework of a telephone consultation is well-suited for selected patients. With respect to patient satisfaction the delegation of a telemedical consultation to an RFA is possible. There is a need for improvement with respect to the vaccination behavior.

## Hintergrund und Fragestellung

Der Lockdown im Rahmen der Pandemie mit dem Schweren Akuten Respiratorischen Syndrom-Coronavirus‑2 (SARS-CoV-2) hatte erstmals zwischen März und Mai 2020 auch Auswirkungen auf die Behandlung ambulanter rheumatologischer Patienten. So erfolgten in den deutschen Kliniken auf Anweisung der Regierung Einschränkungen der elektiven Patientenversorgung vor Ort, um Kapazitäten für die Notfallversorgung freizuhalten [1]. Die Telemedizin rückte als Möglichkeit der kontaktlosen Konsultation in den Fokus. Seitens der Patienten bestanden große Unsicherheit sowie Ängste vor einer Ansteckung mit SARS-CoV‑2, viele Termine wurden daher abgesagt [2, 3]. Aus diesem Grund erfolgte zunehmend der Einsatz telemedizinischer Sprechstunden.

Vor der COVID-19-Pandemie war die Telemedizin in Deutschland noch kaum verbreitet, obwohl ihre Anwendung schon seit 2018 sogar für den Erstkontakt mit Patienten erlaubt ist [4, 5]. Im Jahr 2020 hat die telemedizinische Versorgung aufgrund der COVID-19-Pandemie weltweit einen regelrechten Boom erfahren [1–5]. Auch die Delegation ärztlicher Tätigkeiten in der Rheumatologie an rheumatologische Fachassistenten und Fachassistentinnen (RFA) rückt immer mehr in den Fokus, nicht zuletzt auch, um dem zunehmenden Mangel an Fachärzten gerade in strukturschwachen Regionen entgegenzuwirken [6].

Aktuell laufen verschiedene Studien zum Thema Telemedizin und Delegation, so z. B. in Niedersachsen die ERFASS-Studie, die die Effektivität einer RFA-Sprechstunde evaluieren soll [7].

Ziel unserer Studie war zum einen, die Zufriedenheit von stabilen Patienten aus Klinik und Praxis mit rheumatoider Arthritis (RA), Spondyloarthritis (axSpA) und Psoriasisarthritis (PsA) mit der telemedizinischen Sprechstunde auszuwerten. Zum anderen sollte auch die Zufriedenheit mit Gesprächen mit Arzt oder von einer rheumatologischen Fachassistenz geführten Gesprächen verglichen werden.

## Studiendesign undUntersuchungsmethoden

Stabilen Patienten mit RA, PsA und axSpA wurde bei Terminabsage durch die Praxis bzw. die Hochschulambulanz die Teilnahme an der Studie angeboten. Nach mündlicher Aufklärung und telefonischer Einwilligung wurden die Patienten am ursprünglichen Termin randomisiert entweder von einem Arzt oder einer RFA telefonisch konsultiert. Die Randomisierung wurde in der MHH 1 (RFA): 2 (Arzt) vor Studienbeginn via verschlossener Umschläge durchgeführt, jeder Studienteilnehmer erhielt ein Pseudonym, durch das die Zuordnung der Fragebögen mit den Evaluationsbögen möglich war. In der niedergelassenen Praxis erfolgte die telefonische Konsultation nur ärztlich. Als stabil galten alle Patienten, bei denen während und seit der letzten rheumatologischen Vorstellung keine Änderung der Basistherapie oder Erhöhung der Glukokortikosteroiddosis erforderlich gewesen war und deren krankheitsspezifische Scores (modifizierter CDAI, BASDAI) eine klinische Remission oder nur geringe Krankheitsaktivität anzeigten. Während des Telefongesprächs wurde zunächst die Anamnese standardisiert mittels Fragebogen erhoben und die Krankheitsaktivität mittels modifizierten CDAI oder BASDAI festgestellt. Dabei musste der Patient die Zahl der geschwollenen Gelenke sowie druckempfindlichen Gelenke selbst angeben. Die Schmerzintensität wurde gemäß CDAI/BASDAI anhand einer VAS 0–10 angegeben. Erfolgte das Telefongespräch durch die RFA, wurde ein Arzt von der RFA zusätzlich konsultiert, wenn folgende Faktoren vorlagen: Morgensteifigkeit >45 min, >3 druckschmerzhafte Gelenke, ≥1 geschwollenes Gelenk und/oder Krankheitsaktivität >5 (Skala analog VAS: 1–10). Die Schmerzintensität wurde als separate Variable von 1 (kein Schmerz) bis 10 (maximaler Schmerz) bewertet. Die Einschätzung der klinischen Aktivität wurde ebenfalls als eine eigenständige Variable als VAS von 0 bis 10 erfasst. Im Anschluss wurden standardisierte Fragen hinsichtlich des Impfstatus und der Sorge bezüglich der Corona-Pandemie ausgewertet. Die Sorge hinsichtlich einer COVID-19-Erkrankung wurde in 5 Stufen erfasst, zusätzlich erfolgte die Frage, ob die Patienten ihren Termin aus Sorge vor Ansteckung auch selbst abgesagt hätten (Suppl. Data). Des Weiteren erhielten die Patienten einen pseudonymisierten Evaluationsbogen, angelehnt an den Fragebogen für Arzt-Patienten-Interaktion (FAPI) [8, 9], per Post, um die Patientenzufriedenheit mit dem Telefonat auszuwerten. Die statistische Analyse der Daten erfolgte mittels SPSS® 26 (IBM, Armonk, NY, USA). Alle Korrelationen wurden mittels partieller Korrelation auf Unabhängigkeit von Alter und Diagnose untersucht. Das Signifikanzniveau wurde mittels der nichtparametrischen Kruskal-Wallis- und Mann-Whitney-Tests für unabhängige Stichproben geprüft. Als signifikant wurden *p*-Werte <0,05 definiert.

## Ergebnisse

Insgesamt wurden 112/116 (96 %) Patienten untersucht, nur 4 Patienten lehnten eine Teilnahme ab. Die Hälfte der Patienten (*n* = 56) hatten eine RA, 29,5 % (*n* = 33) litten an einer axSpA und 20,5 % (*n* = 23) an einer PsA. Die Patienten waren überwiegend weiblich (67 %), die Hälfte wurde in einer rheumatologischen Praxis rekrutiert (*n* = 57, 50,9 %), die andere Hälfte in der rheumatologischen Hochschulambulanz der Medizinischen Hochschule Hannover (MHH) (49,1 %). Das mittlere Alter betrug 55,7 Jahre; 23/55 (41,8 %) der Patienten aus der MHH, nur dort wurde die Medikation regelhaft erfasst, erhielten zum Zeitpunkt der Befragung eine Therapie mittels bDMARD, 3 Patienten aus der Hochschulambulanz gaben an, zum Befragungszeitpunkt gar keine Therapie mehr zu erhalten. Alle Patientencharakteristika finden sich in Tab. 1. Den FAPI-Fragebogen haben 28/112 Patienten (25 %) nicht zurückgeschickt. Von den 19 RFA-Befragten schickten 16 (84,2 %) den Fragebogen vollständig beantwortet zurück. Alle zurückgeschickten Evaluationsbögen (*n* = 84) konnten für die FAPI-Auswertung berücksichtigt werden.

**Table 1:** 

	MHH (*n* = 55)	Praxis (*n* = 57)
Weiblich *n* (%)	34 (61,8)	41 (71,9)
Alter (Mittelwert [SD])	61,1 [12,3] Jahre	50,4 [12,6] Jahre
*Diagnosen n (%)*		
Rheumatoide Arthritis	25 (45,5)	31 (54,4)
Psoriasisarthritis	10 (18,2)	13 (22,8)
Spondyloarthritis	20 (36,4)	13 (22,8)
*Basistherapie n (%)*		
Keine Therapie	3 (2,7)	–
csDMARDs	8 (7,1)	–
bDMARDs	23 (20,5)	–
Gesprächslänge min. (Mittelwert [SD])	15,3 [6,1]	12,5 [3,6]
RFA-Anteil *n* (%)	19 (34,5)	–
COVID-Sorgen ja *n* (%)	35 (66)	34 (59,6)
*Terminabsage aufgrund von COVID* ja *n* (%)	14 (26,4)	4 (7)
*Pneumokokkenimpfung *letzte 5 J ja *n* (%)	22 (41,5)	20 (35)
Influenzaimpfung 19/20 ja *n* (%)	28 (52,8)	31 (54,4)
*Morgensteifigkeit* >30 min ja *n* (%)	9 (17,3)	7 (12,3)
*Psoriasis* ja *n* (%)	3 (10,7)	7 (25,9)
*Entzündlicher Rückenschmerz *ja *n* (%)	19 (61,3)	17 (60,7)
*Sehnenschmerz* ja *n* (%)	11 (35,5)	8 (26,9)
*Fersenschmerz* ja *n* (%)	5 (16,1)	0

Basistherapien wurden nur in der MHH erhoben/Prozente beziehen sich auf den Anteil der Patienten, die die Fragen beantwortet haben

Die Telefonsprechstunde wurde mehrheitlich (83 %) durch Ärzte beider Einrichtungen vorgenommen, in der rheumatologischen Praxis gab es keine RFA-Telefonate, in der MHH haben die RFA in 19 Fällen (34,5 %) das Gespräch durchgeführt. Bei 6 (32 %) Patienten wurde zusätzlich der Arzt durch die RFA konsultiert, eine ärztliche Patientenkontaktaufnahme war in keinem dieser Fälle nötig, da die RFA den Sachverhalt klären konnte.

In der Befragung gaben 18 (16,4 %) Patienten an, ihren Termin wegen der COVID-19-Pandemie selbst abgesagt zu haben. Es hätten mehr Patienten der Hochschulambulanz (26,4 %, *n* = 14) ihren Termin abgesagt im Vergleich zu Patienten der niedergelassenen Praxis (7 %, *n* = 4). Weitere 18 (16,4 %) äußerten sehr große Sorgen über das Infektionsgeschehen. Etwa die Hälfte der Patienten gab an, ein bisschen Sorgen zu haben. Insgesamt hatten somit mehr als 60 % der Patienten (*n* = 69) Sorgen, dass sie aufgrund der COVID-19-Pandemie gefährdet sind. Die Patienten mit den größten Sorgen, welche eigenständig ihren Termin abgesagt hätten (*p* = 0,015), waren nicht signifikant älter (*p* = 0,866). Es hätten mehr Patienten aus der Hochschulambulanz aufgrund von Ansteckungssorgen ihren Termin abgesagt als aus der niedergelassenen Praxis. Sorgen wegen der COVID-19-Pandemie waren außerdem mit einer erhöhten Krankheitsaktivität (Anzahl schmerzhafter Gelenke *p* = 0,001/Anzahl geschwollener Gelenke *p* = 0,031/Morgensteifigkeit *p* = 0,022) assoziiert. Es bestand ebenfalls eine Assoziation zu Schmerzen (*p* = 0,009). Insgesamt sorgten sich MHH-Patienten unter Therapie mit einem bDMARD *n* = 17/23 (73,9 %) mehr wegen der COVID-19-Pandemie als die Patienten mit csDMARD *n* = 4/8 (50 %). Alle Korrelationen sind von Alter und Diagnose unabhängig. Der nichtparametrische Kruskal-Wallis-Test zeigte ebenfalls eine Korrelation von gesteigerten Sorgen wegen einer möglichen COVID-19-Infektion und der Anzahl druckschmerzhafter Gelenke.

In Bezug auf Impfungen gaben 38,2 % der Patienten an, in den letzten Jahren gegen Pneumokokken geimpft worden zu sein, 53,6 % der Patienten wurden in der Saison 2019/2020 gegen Influenza geimpft. Das durchschnittliche Alter der geimpften Personen war im Vergleich zu den nicht geimpften Patienten signifikant höher (Pneumokokken 59,3 vs. 53,2 Jahre/Influenza 58,7 vs. 51,9 Jahre). Dies gilt sowohl für die Impfung gegen Pneumokokken (*p* = 0,033) als auch für die Impfung gegen Influenza (*p* = 0,012).

Patienten, die mit einem Biologikum therapiert wurden, waren zu je 39,1 % gegen Pneumokokken und gegen Influenza geimpft. Die mit csDMARDs behandelten Patienten waren zu 62,5 % gegen Pneumokokken und zu 87,5 % gegen Influenza geimpft. Die geringe Patientenanzahl lässt hier jedoch keine Signifikanzberechnungen zu. Das Impfverhalten der Patienten in der Hochschulambulanz unterschied sich nicht von den Patienten in der Praxis.

Bezüglich der Krankheitsaktivität gaben 53,2 % (*n* = 58) eine Morgensteifigkeit >30 min und 18,2 % der Patienten eine Psoriasisaktivität an, diese war definiert als aktueller Nachweis von Hautmanifestation, die bei 80 % der PsA-Patienten vorlag; 61 % (*n* = 36) der Patienten klagten über entzündlichen Rücken-, 32,8 % (*n* = 19) über Sehnen- und 8,6 % (*n* = 5) über Fersenschmerz.

In Tab. 2 sind Mittelwerte der schmerzhaften und geschwollenen Gelenke sowie der Schmerzintensität und der patienteneigenen Einschätzung des Krankheitszustandes dargestellt.

**Table 2:** 

	Anzahl schmerzhafter Gelenke	Anzahl geschwollener Gelenke	Eigene Einschätzung des Zustandes	Schmerzintensität
Mittelwert	2,42	0,90	3,86	4,32
*N*	104	106	96	96
Std.-Abweichung	4,543	2,453	2,315	2,577

Die telefonische Gesprächsdauer bei Patienten mit sehr ausgeprägten COVID-19-Sorgen war im Mittel signifikant länger (14,4 min, SD 3,8) als bei jenen, die keinerlei COVID-19-Sorgen angegeben hatten (12,1 min, SD 5,2) (*p* = 0,046).

Die Gespräche in der Hochschulambulanz waren im Durchschnitt etwas länger als die aus der Praxis (Hochschulambulanz 15,3 min, SD 6,1 vs. Praxis 12,5 min, SD 3,6; *p* = 0,015). RFA-Gespräche und Arztgespräche unterschieden sich nicht hinsichtlich der Gesprächsdauer (durchschnittlich 14,3 vs. 14,1 min). Auch die Patientenzufriedenheit war letztlich unabhängig von der Gesprächsdauer in beiden Einrichtungen sehr gut (Mittelwert 4,3/5,0 modifizierter FAPI).

Mittels modifizierten FAPI-Scores wurden nach dem Telefongespräch die Patientenwahrnehmung und die Zufriedenheit des Gespräches evaluiert. Patienten mit einer hohen Schmerzintensität waren tendenziell zum Zeitpunkt des Gesprächs am unzufriedensten, unabhängig von der Länge des Telefonats oder wer das Gespräch geführt hat (*p* = 0,051). Diese Tendenz stellte sich nicht bei der Krankheitsaktivität dar (*p* = 0,68).

Bei detaillierter Analyse des FAPI wurde dieser in 2 Kategorien unterteilt. Die eine beschrieb mit den Items 3 bis 6, 9 und 13 die patientenbezogene und empathische Grundhaltung, die zweite mit den Items 2, 7, 10 und 11 die Problemklärung durch den Arzt [9]. Dabei zeigte sich ein signifikanter Zusammenhang der patientenbezogenen empathischen Grundhaltung mit der Schmerzintensität: je höher der Schmerz, desto niedriger die Patientenzufriedenheit (*p* = 0,036).

## Diskussion

Diese Studie zeigt, dass die telefonische Sprechstunde bei stabilen Patienten sowohl in der Praxis als auch in einem universitären Ambulanzzentrum gut angenommen wurde. Die Patienten waren überwiegend sehr zufrieden mit dem Telefonat, unabhängig davon, ob das Gespräch durch eine RFA oder einen Arzt durchgeführt wurde. Unzufriedenheit war assoziiert mit Schmerzen zum Zeitpunkt der Konsultation und war unabhängig von der Krankheitsaktivität.

Es zeigte sich in den Ergebnissen, dass Patienten, die mit einem Biologikum eingestellt sind, besorgter waren hinsichtlich einer COVID-19-Erkrankung. Aktuell liegen bezüglich der Prognose von COVID-19-Infektionen bei rheumatologischen Erkrankungen kontroverse Daten vor [10]. Bei kritischen COVID-19-Verläufen werden auch immunsupprimierende Medikamente wie Dexamethason [11] eingesetzt und waren in einigen Studien mit einer besseren Prognose assoziiert. Die Datenlage ist diesbezüglich insgesamt aber noch nicht eindeutig, und der Einsatz immunsupprimierender Medikamente wird immer wieder kontrovers diskutiert [12]. Aktuell kommt z. B. Tocilizumab in Großbritannien regelhaft zum Einsatz. Positive Auswirkungen wurden auch für Kinaseinhibitoren gesehen [13]. Patienten mit rheumatologischen Grunderkrankungen zeigten in einer amerikanischen Registerstudie mit 300 eingeschlossenen Patienten keine besonders schweren Verläufe von COVID-19-Erkrankungen im Vergleich zur Normalbevölkerung [14]. Insbesondere die Therapie mit Biologika war nicht mit schwereren Verläufen assoziiert, nur für Patienten mit Steroiddosen >5 mg/Tag sowie Komorbiditäten (Diabetes mellitus, kardiovaskuläre Erkrankungen) ergab sich ein erhöhtes Risiko für Hospitalisierungen [14, 15]. Es ließen sich bei Patienten mit rheumatologischen Erkrankungen häufiger Risikokomorbiditäten wie arterielle Hypertonie, Adipositas oder aktiver Nikotinkonsum finden [16].

Patienten, die mit einem Biologikum behandelt wurden, ließen sich interessanterweise weniger häufig impfen, ggf. ist dies mit dem etwas jüngeren Alter dieser Patienten assoziiert [17, 18]. Doch die Impfraten sind trotz regelmäßiger Empfehlung zur Impfung mittels Gesprächen, Arztbriefen und Mitgabe von Informationsblättern sowohl in der Hochschulambulanz als auch in der Praxis alarmierend niedrig.

Es ist zu diskutieren, ob eine Impfung der Patienten in der rheumatologischen Betreuung erfolgen sollte. Gerade unter Berücksichtigung der aktuellen Pandemie ist eine Impfung gegen Pneumokokken und Influenza besonders wichtig [19]. Seit Kurzem besteht die Möglichkeit, auch gegen COVID-19 zu impfen. Diese Impfung wird von der Fachgesellschaft für Rheumatologie ebenfalls ausdrücklich für alle Patienten mit rheumatologischen Grunderkrankungen auch unter immunsuppressiver Therapie empfohlen [19].

RFA-Sprechstunden sind bei stabilen Patienten eine wertvolle Möglichkeit, gerade in unterversorgten Gebieten eine gute und regelmäßige Betreuung von Patienten mit rheumatologischen Erkrankungen zu gewährleisten. Eine große Hürde stellt zum aktuellen Zeitpunkt noch die fehlende Vergütung dieser RFA-Sprechstunden dar, die ein solches Konzept noch unattraktiv macht. Insgesamt stellt die Vergütung der Telemedizin noch einen großen Konfliktpunkt dar. Zwar wird die Telemedizin per se von einem großen Anteil der Ärzte unterstützt, insbesondere der Ärzte unter 40 Jahren, die Vergütung wird allerdings von einem großen Teil als unzureichend eingeschätzt [2, 3, 5]. Dazu tragen zum aktuellen Zeitpunkt die fehlende Vergütung der telemedizinischen Erhebung von Krankheitsaktivitätsscores und die fehlenden Einnahmen durch die Laborkontrollen bei. Derzeit können für die Durchführung einer telefonischen Konsultation, die durchschnittlich mehr als 13 min dauerte, nur weniger als 10 € abgerechnet werden. Für eine Videosprechstunde kann die Grundpauschale mit 25 % Abschlag angesetzt werden, ggf. ergänzt durch limitierte Zuschläge. Auch dies kann kaum die Kosten dieser Versorgungsart decken.

Die Gesprächsdauer des Telefonats unterschied sich signifikant zwischen Hochschulambulanz und Praxis(15,32 min vs. 12,5 min). Der Unterschied ist am ehesten mit dem höheren Patientenaufkommen und den auch insgesamt kürzeren Konsultationszeiten in der Niederlassung im Vergleich zu Hochschulambulanzen zu erklären, wo häufig komplexere und schwerere Krankheitsfälle behandelt werden. Die längsten Gespräche wurden mit den Patienten mit den meisten Sorgen geführt. Es sind folglich nicht unbedingt die Rheumapatienten mit Krankheitsaktivität, die am zeitaufwendigsten in der telemedizinischen Versorgung sind, sondern v. a. die Patienten mit vielen Sorgen bzw. oft auch Schmerzen, die nicht durch Krankheitsaktivität bedingt sind. Dieser Aspekt sollte nochmals untersucht und bestätigt werden, da die erhöhte zeitliche Belastung bei der Behandlung von Patienten mit chronischen Schmerzen in der ambulanten rheumatologischen Versorgung, insbesondere in unterversorgten Regionen, eine zusätzliche Belastung darstellen könnte.

Limitiert ist unsere Analyse durch die recht geringe Patientenanzahl und den fehlenden Vergleich zur Patientenzufriedenheit mit der Präsenzsprechstunde, denn auch in der Präsenzsprechstunde ist ggf. die Zufriedenheit der Patienten mit den meisten Schmerzen eher als geringer einzuschätzen.

## Fazit für die Praxis

Zusammenfassend kann festgehalten werden, dass die Telemedizin auch außerhalb von Pandemiezeiten eine wertvolle Ergänzung zu den klassischen Präsenzterminen, insbesondere für stabile Patienten darstellen kann. Die Patientenzufriedenheit mit der Telefonsprechstunde war hoch, sowohl bei ärztlicher Konsultation als auch durch eine RFA durchgeführte Sprechstunde, unabhängig von der Länge des Gesprächs. Folglich ist diese Delegation an eine RFA zu diskutieren. Doch diese Arbeit müsste sich dann auch in der Abrechnung wiederfinden, aktuell werden delegierte RFA-Tätigkeiten kaum vergütet, und auch telemedizinische Sprechstunden sind für rheumatologische Sprechstunden finanziell meist unattraktiv. Durch standardisierte Befragungen können erfahrene RFAs eine sichere und qualitativ hochwertige Sprechstunde durchführen. Durch sog. Red-Flag-Antworten kann sichergestellt werden, dass bei möglicher Krankheitsaktivität der Arzt zusätzlich konsultiert wird. Hinsichtlich des Impfverhaltens müssen wir sowohl in der Hochschulambulanz als auch in der Niederlassung besser werden. Verschiedene Möglichkeiten, wie z. B. Impfen beim Rheumatologen, bessere Absprachen mit Hausärzten oder eine Impfpassprüfung beim Rheumatologen sind zu diskutieren.

## Supplementary Information




