# Supplementary material for: Care of rheumatology patients during the lockdown in early 2020: Telemedicine, delegation, patient satisfaction and vaccination behavior
Source: Z Rheumatol. 2021 May 11;81(2):157–63. [Article in German] doi: 10.1007/s00393-021-01005-3 (PMC8111653; doi:10.1007/s00393-021-01005-3)
Supplement: Supplementary file 1 [file 393_2021_1005_MOESM1_ESM.pdf]

DATUM: **Telefongespräch geführt durch:** **RFA** **ARZT/ÄRZTIN**

Patientencode: **Praxis/MHH**

Patientenfragebogen RA: zutreffendes bitte einkreisen

COVID:

1. Hätten Sie Ihren Termin aufgrund der COVID Pandemie selbst abgesagt? JA NEIN
2. Haben Sie Sorge, dass Sie aufgrund der COVID Pandemie besonders gefährdet sind?

   Ja sehr ein bisschen keine Meinung eher nicht überhaupt nicht

3. Sind Sie in den letzten 5-6 Jahren gegen Pneumokokken (Lungenentzündung) geimpft worden? JA NEIN
4. Sind Sie aktuell gegen Grippe (Influenza) geimpft? JA NEIN

Krankheitsaktivität:

1. Haben Sie in den letzten 4 Wochen Prednisolon (Cortison) erhöht? JA NEIN
2. Besteht eine Morgensteifigkeit der Gelenke? JA NEIN

   Falls ja, wie lange hält diese an?

   15 min 30 min 45 min 60 min 90 min 120 min

   (über 45 min. bitte Arzt kontaktieren)
3. Wie viele Gelenke schmerzen bei Druck?

   (mehr als 3 bitte Arzt kontaktieren)
4. Sind aktuell Gelenke geschwollen?

   (mehr als 1 bitte Arzt kontaktieren)

   Anzahl kleine Gelenke (Finger/Zehen): Anzahl große Gelenke:
5. Einschätzung klinische Aktivität (1 keine – 10 extrem) (mehr als 5 bitte Arzt kontaktieren):
6. Schmerzintensität (1 keine – 10 extrem):

Für RFA:

Rücksprache mit Arzt erfolgt: JA NEIN falls ja: Gesprächsdauer: min

Arzt musste Patient kontaktieren: JA NEIN falls ja: Gesprächsdauer: min

Therapeutische Konsequenz:

Therapieänderung JA NEIN

Falls ja: Prednisolonerhöhung Basistherapiewechsel andere

DATUM: **Telefongespräch geführt durch:** **RFA** **ARZT/ÄRZTIN**

Patientencode: **Praxis/MHH**

Patientenfragebogen PsA/SpA: zutreffendes bitte einkreisen

COVID:

5. Hätten Sie Ihren Termin aufgrund der COVID Pandemie selbst abgesagt? JA NEIN
6. Haben Sie Sorge, dass Sie aufgrund der COVID Pandemie besonders gefährdet sind?

   Ja sehr ein bisschen keine Meinung eher nicht überhaupt nicht

7. Sind Sie in den letzten 5-6 Jahren gegen Pneumokokken (Lungenentzündung) geimpft worden? JA NEIN
8. Sind Sie in der Saison 2019/2020 gegen Grippe/Influenza geimpft? JA NEIN

Krankheitsaktivität:

7. Haben Sie in den letzten 4 Wochen Prednisolon (Cortison) erhöht? JA NEIN
8. Besteht eine Morgensteifigkeit der Gelenke? JA NEIN

   Falls ja, wie lange hält diese an?

   15 min 30 min 45 min 60 min 90 min 120 min

   (über 45 min. bitte Arzt kontaktieren)
9. Wie viele Gelenke schmerzen bei Druck?

   (mehr als 3 bitte Arzt kontaktieren)
10. Sind aktuell Gelenke geschwollen?

    (mehr als 1 bitte Arzt kontaktieren)

    Anzahl kleine Gelenke (Finger/Zehen): Anzahl große Gelenke:
11. Einschätzung klinische Aktivität (1 keine – 10 extrem) (mehr als 5 bitte Arzt kontaktieren):
12. Schmerzintensität (1 keine – 10 extrem):
13. Ist eine evtl. vorhandene Psoriasis/Schuppenflechte aktuell aktiv? JA NEIN
14. Besteht derzeit ein seit mind. 1 Woche anhaltender Rückenschmerz?

    Ist dessen Schmerzmaximum morgens? JA NEIN

    Bessert sich der Rückenschmerz bei Bewegung JA NEIN
15. Bestehen Schmerzen an Sehnenansätzen? JA NEIN
16. Haben Sie aktuell Schmerzen an den Fersen? JA NEIN

Für RFA

Rücksprache mit Arzt erfolgt: JA NEIN falls ja: Gesprächsdauer: min

Arzt musste Patient kontaktieren: JA NEIN falls ja: Gesprächsdauer: min

Therapeutische Konsequenz:

Therapieänderung JA NEIN

Falls ja: Prednisolonerhöhung Basistherapiewechsel andere

Telefonat am: durch: RFA Arzt/Ärztin

Länge des Telefonats:

Patientencode: Grunderkrankung:

Liebe/r Patient*in,

bitte füllen Sie, wie besprochen, den Fragebogen aus und schicken ihn zur Evaluation unserer Telefonsprechstunde an uns zurück. Vielen Dank!

	trifft nicht zu	trifft teils zu	trifft zu	trifft sehr zu	trifft ganz genau zu
1. Der/Die Anrufer*in schien ein echtes Interesse an meinen Problemen zu haben.	1	2	3	4	5
2. Der/Die Anrufer*in hat mich ausführlich über die vorhandenen Behandlungsmöglichkeiten informiert.	1	2	3	4	5
3. Ich hatte das Gefühl, dass ich dem/der Anrufer*in auch sehr persönliche Probleme hätte anvertrauen können.	1	2	3	4	5
4. Der/Die Anrufer*in hat alle Behandlungsmaßnahmen gemeinsam mit mir festgelegt.	1	2	3	4	5
5. Die Erklärungen der/der Anrufer*in waren für mich sehr verständlich.	1	2	3	4	5
6. Der/Die Anrufer*in hat sich genügend Zeit für mich genommen.	1	2	3	4	5
7. Der/Die Anrufer*in hat mit mir ausführlich über die Risiken und Nebenwirkungen der Behandlung gesprochen (falls Therapiewechsel).	1	2	3	4	5
8. Meine Probleme und Nöte wurden von dem/der Anrufer*in verstanden und ernst genommen.	1	2	3	4	5
9. Der/Die Anrufer*in hat das Möglichste getan, um mich zu beruhigen.	1	2	3	4	5
10. Der/Die Anrufer*in hat sich nach den Auswirkungen der Erkrankung auf meinen Alltag erkundigt.	1	2	3	4	5
11. Der/Die Anrufer*in gab mir genügend Möglichkeiten, meine Schwierigkeiten und Probleme zu schildern.	1	2	3	4	5
12. Der/Die Anrufer*in respektiert es, wenn ich eine abweichende Meinung zur Behandlung habe.	1	2	3	4	5
13. Der/Die Anrufer*in hat mich ausführlich über meine aktuellen Beschwerden befragt.	1	2	3	4	5
